# A sensitive and bright single-cell resolution live imaging reporter of Wnt/ß-catenin signaling in the mouse

**DOI:** 10.1186/1471-213X-10-121

**Published:** 2010-12-21

**Authors:** Anna Ferrer-Vaquer, Anna Piliszek, Guangnan Tian, Robert J Aho, Daniel Dufort, Anna-Katerina Hadjantonakis

**Affiliations:** 1 Developmental Biology Program, Sloan-Kettering Institute, New York, NY, USA; 2 Department of Obstetrics and Gynecology, Division of Experimental Medicine, McGill University Health Center, Royal Victoria Hospital, Montreal, QC, Canada

## Abstract

**Background:**

Understanding the dynamic cellular behaviors and underlying molecular mechanisms that drive morphogenesis is an ongoing challenge in biology. Live imaging provides the necessary methodology to unravel the synergistic and stereotypical cell and molecular events that shape the embryo. Genetically-encoded reporters represent an essential tool for live imaging. Reporter strains can be engineered by placing *cis*-regulatory elements of interest to direct the expression of a desired reporter gene. In the case of canonical Wnt signaling, also referred to as Wnt/β-catenin signaling, since the downstream transcriptional response is well understood, reporters can be designed that reflect sites of active Wnt signaling, as opposed to sites of gene transcription, as is the case with many fluorescent reporters. However, even though several transgenic Wnt/β-catenin reporter strains have been generated, to date, none provides the single-cell resolution favored for live imaging studies.

**Results:**

We have placed six copies of a TCF/Lef responsive element and an *hsp68 *minimal promoter in front of a fluorescent protein fusion comprising human histone H2B to GFP and used it to generate a strain of mice that would report Wnt/β-catenin signaling activity. Characterization of developmental and adult stages of the resulting *TCF/Lef:H2B-GFP *strain revealed discrete and specific expression of the transgene at previously characterized sites of Wnt/β-catenin signaling. In support of the increased sensitivity of the *TCF/Lef:H2B-GFP *reporter, additional sites of Wnt/β-catenin signaling not documented with other reporters but identified through genetic and embryological analysis were observed. Furthermore, the sub-cellular localization of the reporter minimized reporter perdurance, and allowed visualization and tracking of individual cells within a cohort, so facilitating the detailed analysis of cell behaviors and signaling activity during morphogenesis.

**Conclusion:**

By combining the Wnt activity read-out efficiency of multimerized TCF/Lef DNA binding sites, together with the high-resolution imaging afforded by subcellularly-localized fluorescent fusion proteins such as H2B-GFP, we have created a mouse transgenic line that faithfully recapitulates Wnt signaling activity at single-cell resolution. The *TCF/Lef:H2B-GFP *reporter represents a unique tool for live imaging the *in vivo *processes triggered by Wnt/β-catenin signaling, and thus should help the formulation of a high-resolution understanding of the serial events that define the morphogenetic process regulated by this signaling pathway.

## Background

Wnt signaling is a key, evolutionarily conserved, cellular signal transduction pathway required and reiteratively used for diverse biological functions. Precise regulation of pathway activity is required for proper embryonic development, and in adulthood, for tissue homeostasis. By contrast, impaired Wnt signaling activity can lead to embryonic defects and disease progression. Wnt proteins encompass a large family of secreted glycoproteins that trigger their outcome through different downstream cascades, among them the canonical Wnt/ß-catenin pathway, which activates transcription of target genes by the stabilization and nuclear localization of ß-catenin, a transcriptional co-activator protein.

In the absence of ligand, cytoplasmatic ß-catenin is phosphorylated and targeted for degradation by a protein complex consisting of the scaffolding proteins Axin, APC and the kinase GSK3ß. Once phosphorylated, ß-catenin is recognized by the ubiquitin ligase Trcp, which targets it for proteasomal degradation. Upon binding of the Wnt ligand to the receptor complex formed by Frizzled (Fz) and LRP5/6, Dishevelled (Dvl) is recruited by Fz leading to LRP5/6 phosphorylation and Axin recruitment. Loss of Axin from the degradation complex dismantles the complex and releases ß-catenin. Once stabilized, ß-catenin translocates to the nucleus. As a transcriptional coactivator, ß-catenin together with the T cell-specific transcription factor/lymphoid enhancer-binding factor 1 (TCF/Lef) family of transcription factors induces the transcription of downstream genes (reviewed in [1-3]).

Over the past fifteen years several transgenic mouse strains have been established to monitor Wnt/ß-catenin pathway activity during development, homeostasis and disease progression (reviewed in [4]). These reporter constructs are generally derived from the *TOPFLASH *design [5]. They consist of a series of multimerized DNA binding sites for TCF/Lef (TCF/Lef_n_), which together with a minimal promoter (*promoter_min)_*, drive expression of a reporter gene. Thus, in principle, such *TCF/Lef_n_-promoter_min_:reporter *constructs label cells that are actively transducing a Wnt signal.

As a validation of their utility, several variant Wnt/ß-catenin reporter mouse strains have been characterized, are readily available and have been used to determine Wnt signaling status in a broad spectrum of applications. First generation constructs usually comprised a LacZ reporter (e.g. [6]), whereas later generation versions provided a quantifiable readout and promoted live imaging applications by incorporating fluorescent protein reporters such as GFP [7,8]. Even so, because of their robust expression and resistance to fixation, LacZ reporters have often been preferable for higher resolution analysis of sectioned tissues. By contrast, native fluorescent proteins, even though desirable for live imaging applications, usually cannot be visualized at single-cell resolution, and often do not withstand fixation and post-processing. Thus, none of the existing *TCF/Lef_n_-promoter_min_:reporter *constructs, or derivative mouse strains, facilitate single-cell resolution imaging of Wnt/ß-catenin pathway activity that can be quantified in live as well as in fixed tissues.

We therefore sought to generate an improved, third generation, Wnt/ß-catenin reporter, that would incorporate a bright fluorescent reporter which could be live imaged at single-cell resolution and also quantified, but which would withstand fixation and therefore could also be visualized in tissue sections. To do so, we designed a reporter construct that combined the Wnt/ß-catenin signaling read-out efficiency of multimerized TCF/Lef DNA binding sites with the single-cell resolution and quantifiable reporter expression afforded by fluorescent histone fusions. Fluorescent proteins fused to histones, for example human histone H2B, are localized to the nucleus because they remain bound to chromatin, and as such allow the visualization and tracking of individual cells. H2B fusions also provide details of cell divisions, including the plane of division and identification of daughter cells. They also reveal nuclear fragmentation which is often associated with cell death [9,10]. We placed the H2B-GFP cassette under the control of six *TCF/Lef *response elements and the *hsp68 *minimal promoter in a configuration identical to previously reported *TCF/Lef-LacZ *reporter mice [11]. We then used this construct to generate a derivative *TCF/Lef:H2B-GFP *mouse strain.

We recovered several founder transgenic lines which exhibited equivalent expression, demonstrating that *TCF/Lef:H2B-GFP *reporter expression was independent of integration site. Characterization of the *TCF/Lef:H2B-GFP *strain of mice revealed bright single-cell resolution reporter expression that spatio-temporally recapitulated *TCF/Lef-LacZ *reporter expression during mouse embryonic development. Moreover, given its improved sensitivity, the *TCF/Lef:H2B-GFP *strain revealed additional sites of reporter expression, in the visceral endoderm and epiblast of the pre-gastrula stage mouse embryo, tissues suggested through genetic and expression analyses to possess active Wnt/ß-catenin signaling, that is not reflected by existing Wnt/ß-catenin signaling reporters.

In summary, we have generated a transgenic mouse strain that serves as a quantitative, non-invasive single-cell resolution read-out of Wnt/ß-catenin signaling in the mouse. The *TCF/Lef:H2B-GFP *reporter currently represents an improved tool for imaging the *in vivo *processes triggered by canonical Wnt signaling pathway activation.

## Results and Discussion

### Generation of TCF/Lef:H2B-GFP reporter mice

**Figure 1:**
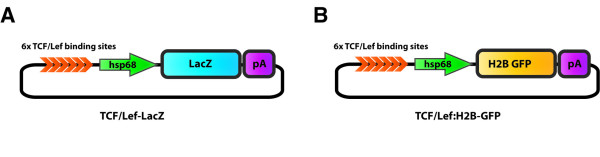
***TCF/Lef:H2B-GFP *construct design**. *TCF/Lef-LacZ *(A) and *TCF/Lef:H2B-GFP *(B) construct design. Each construct consists of six *TCF/Lef *response elements, which together with the *hsp68 *minimal promoter, drive the expression of *ß-galactosidase *in the *TCF/Lef-LacZ *construct or *GFP *in the *TCF/Lef:H2B-GFP *construct.

Different promoter elements used in already characterized LacZ reporter lines were considered as drivers for H2B-GFP reporter expression after Wnt signaling activation. We opted for both the BAT-gal and the *TCF/Lef-LacZ *reporter lines, since both have been extensively used in recent years as reliable read-outs of canonical Wnt activity. In the BAT-gal transgene construct, the promoter elements driving reporter expression consist of 7 TCF/Lef binding sites together with a 130 bp fragment containing the minimal promoter-TATA box of the gene *siamois *[12]. On the other hand, the *TCF/Lef-LacZ *construct was generated by fusing six *TCF/Lef *response elements upstream of the *hsp68 *minimal promoter (Figure 1A) [11].

We subsequently cloned the *TCF/Lef *response elements and minimal promoter of each reporter in front of H2B-GFP to create the final construct. Transgenic mouse lines were generated from both constructs. However, since the *TCF/Lef-siamois:H2B-GFP *transgenic animals gave no readily detectable fluorescent signal, only the *TCF/Lef-hsp68:H2B-GFP *founder lines were considered for further study, and from now on are referred as *TCF/Lef:H2B-GFP *strain (Figure 1B).

**Figure 2:**
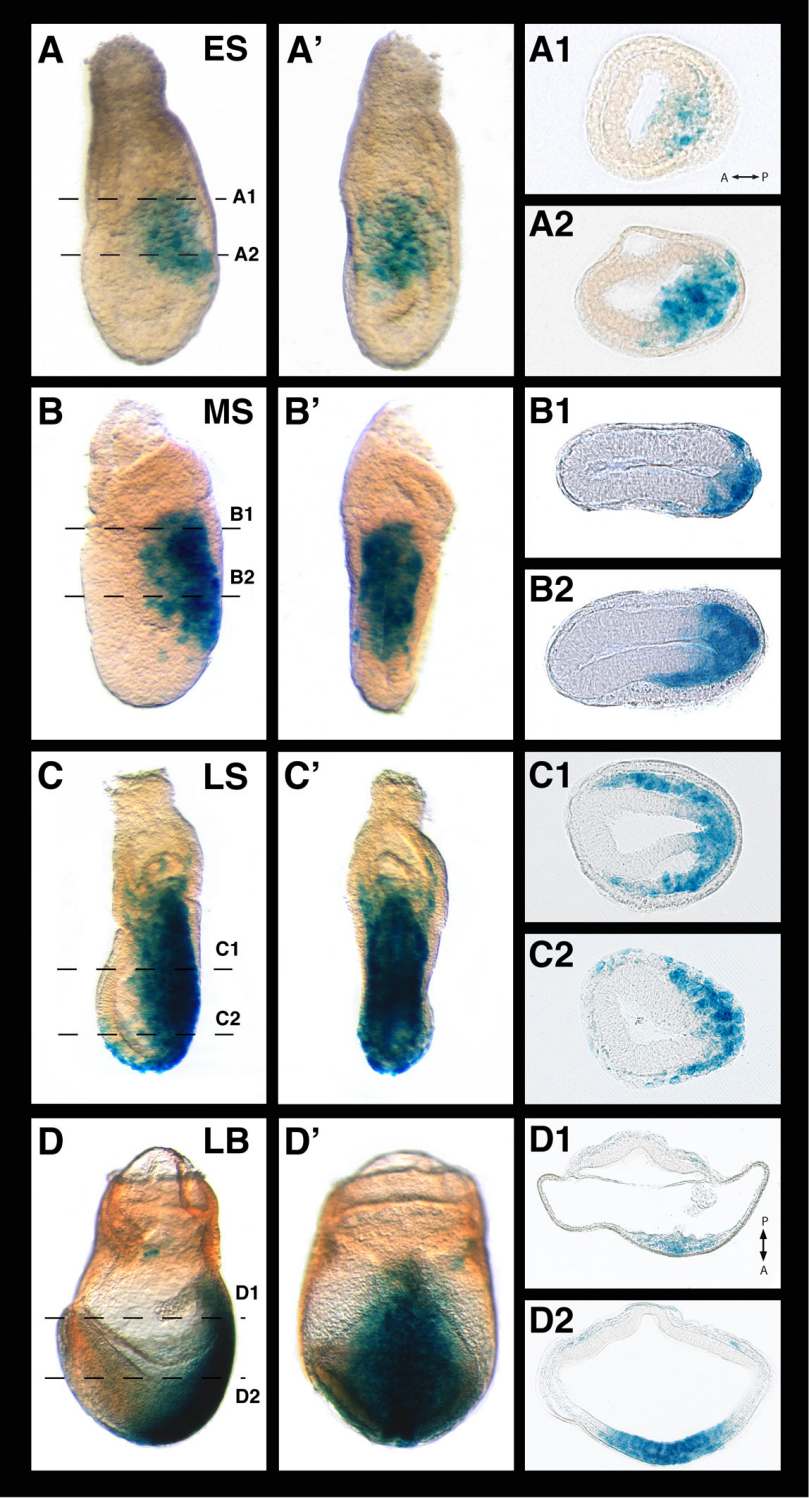
**Charaterization of the *TCF/Lef-LacZ *transgene expression at gastrulation**. Lateral (A) and posterior (A') views of an early streak embryo stained for ß-galactosidase activity. Transverse sections (A1, A2) show LacZ staining at the posterior part of the embryo marking streak initiation. Lateral and posterior views of a mid-streak (B, B') and late streak (C, C') embryos and transverse sections through them (B1, B2, C1, C2 respectively) showing LacZ staining in the primitive streak and wings of mesoderm. Lateral (D) and posterior (D') views of a late bud embryo positive for the transgene and sections through it (D1, D2) along the indicated planes. ES, early streak; LB, late bud; LS, late streak; MS, mid-streak.

**Figure 3:**
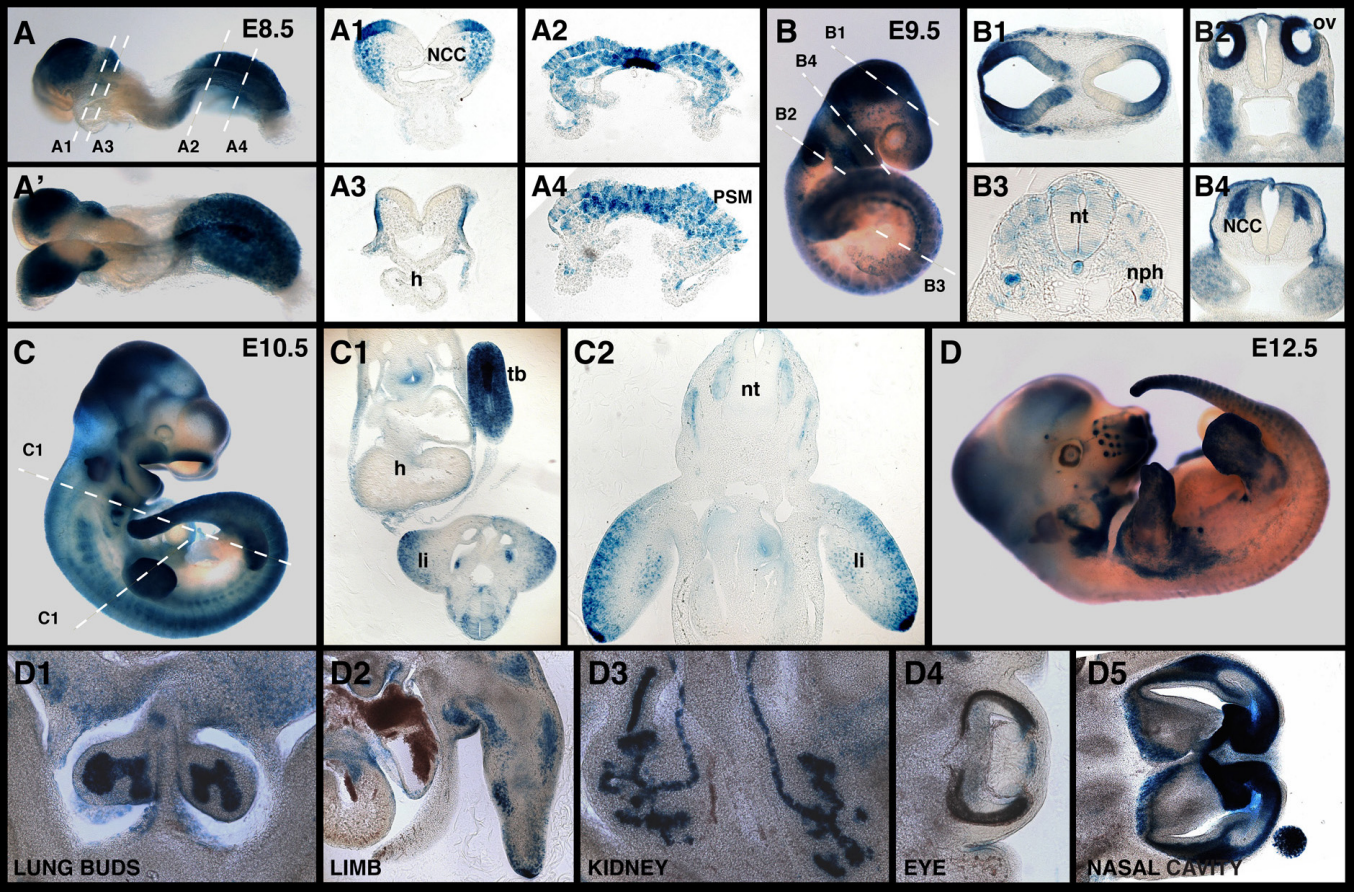
**Expression of the *TCF/Lef-LacZ *transgene from E8.5 to E12.5**. Lateral (A) and dorsal (A') views of an E8.5 embryo stained for ß-galactosidase activity. Sections through the same embryo show staining in the NCC (A1), the presomitic mesoderm (A2, A4), notochordal plate (A2) and ectoderm (A3). (B) Lateral view of an E9.5 embryo positive for the transgene and sections through it, revealing expression in the brain (B1), NCC (B2, B4), notochord, nephric duct and faintly in the neural tube and somites (B3). (C) Expression of the transgene in an E10.5 embryo and transverse sections (C1, C2) along the indicated planes show staining in the limb, neural tube, gut and tail bud. (D) Whole mount view of an E12.5 embryo stained for ß-galactosidase activity. Sections through the same embryo reveal staining in the lung buds (D1), limb mesenchyme (D2), kidney tubules (D3), eye (D4) and nasal cavity (D5). h, heart; li, limb; NCC, neural crest cells; nph; nephric duct; nt, neural tube; ov, otic vesicle; PSM, presomitic mesoderm; tb, tail bud.

**Figure 4:**
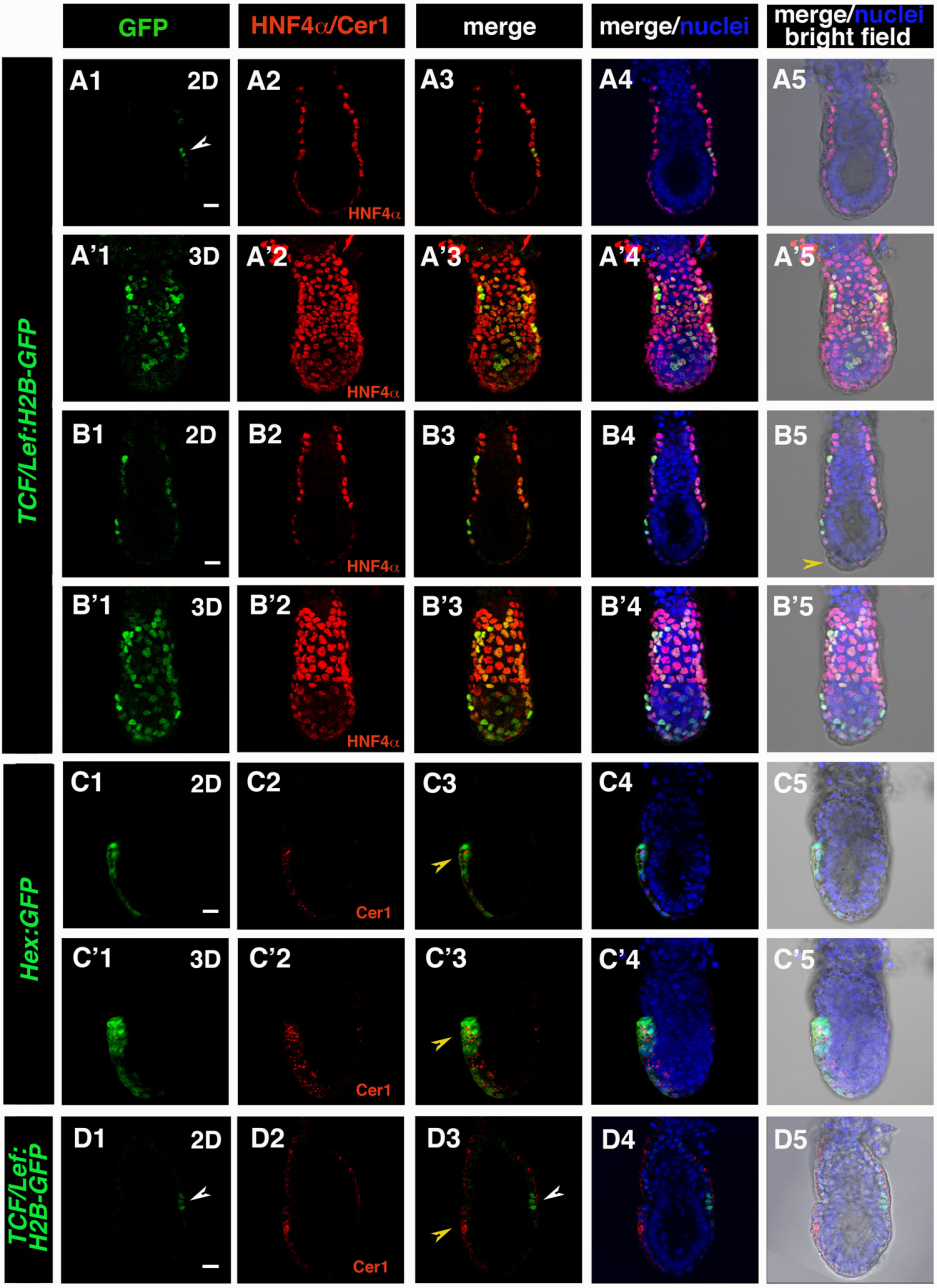
***TCF/Lef:H2B-GFP *reporter expression in pre-streak stage embryos**. Laser scanning confocal images of (A-B, D) E5.5-E5.75 *TCF/Lef:H2B-GFP *and (C) *Hex:GFP *embryos. (A, B) H2B-GFP in *TCF/Lef:H2B-GFP *embryos was localized to the VE in E5.5 embryos, and colocalized with VE marker HNF4α. (A, A') Prior to DVE specification GFP is detected in a subset of VE cells. (B, B') After initiation of DVE migration (B5, yellow arrowhead) GFP is localized in the majority of VE cells, showing varying levels of GFP expression. (C) Cer1 as a marker of AVE- Cer1 localization overlaps with AVE-specific GFP reporter in *Hex:GFP *embryos (C3, C'3; yellow arrowhead) (D) A group of GFP positive cells (white arrowhead) localized posteriorly (A1, D1), in relation to Cer1 (AVE, yellow arrowhead) localized anteriorly (D3; AVE, yellow arrowhead). Each row represents one embryo. Panels depict single optical sections (A, B, C, D), or 3D reconstructions (A', B', C') of confocal z-stacks. Green, TCF/Lef:H2B-GFP, Hex:GFP; red, Cer1, HNF4α; blue, Hoechst. Scale bar: 20 μm.

To establish the specificity of reporter expression, and determine that we did not observe any position or copy-number dependent variability, we confirmed that two independent *TCF/Lef:H2B-GFP *(F_0_) founder animals produced identical patterns of reporter expression in F_1 _progeny. We then compared the pattern of expression of the *TCF/Lef:H2B-GFP *reporter with that observed in stage-matched embryos of the related *TCF/Lef-LacZ *strain (Figure 2 and 3). Overall, the H2B-GFP-based reporter confirmed all sites of expression observed with the LacZ reporter at all stages and in all tissues examined. This validated the specificity of our *TCF/Lef:H2B-GFP *reporter as a single-cell resolution live imaging read-out of canonical Wnt signaling activity in mouse embryos and adult tissues. Moreover, as we had predicted, the *TCF/Lef:H2B-GFP *reporter revealed cells undergoing active Wnt/ß-catenin signaling within the epiblast and visceral endoderm of the early postimplantation stage embryo (Figure 4). Genetic analysis and marker expression data have supported a role for Wnt/ß-catenin signaling in these tissues of the early embryo, but these sites have not been substantiated using previous generation Wnt/ß-catenin signaling reporter strains.

### Failure to detect TCF/Lef:H2B-GFP reporter expressing cells at preimplantation stages of embryonic development

Considerable debate has surrounded the issue of Wnt/ß-catenin signaling at preimplantation stages of mouse development, and specifically at the blastocyst stage. In support of a transcriptional read-out of canonical Wnt signaling, previous reports have suggested possible transient nuclear-localization of ß-catenin in a minor population of cells at the blastocyst stage. In our hands and with our *TCF/Lef:H2B-GFP *reporter, which we believe to exhibit increased sensitivity over existing reporter strains, we were unable to detect convincing reproducible nuclear-localized GFP fluorescence at any stage of preimplantation development in either successively staged or time-lapse imaged embryos. We take this to suggest that either the *TCF/Lef:H2B-GFP *reporter is not sufficiently sensitive, or that any Wnt/ß-catenin pathway signaling response is non-transcriptional.

### TCF/Lef:H2B-GFP reporter reveals sites of Wnt/ß-catenin signaling in the early postimplantation embryo not previously detected with reporter strains

At the late blastocyst stage (E4.5) the embryo implants into the maternal uterus. This event is followed by lineage expansion, which results in formation of a cup-shaped egg cylinder. Close apposition of epiblast, extraembryonic endoderm and visceral endoderm (VE) facilitates signaling cross-talk between these layers, leading to the formation of the distal visceral endoderm (DVE), migration of the anterior visceral endoderm (AVE) and establishment of the anterior-posterior axis of the embryo [13]. Canonical Wnt signaling has been implicated in both formation of anterior-posterior axis and AVE migration. Specifically, a population of cells which are proposed to actively migrate within the VE epithelium, a morphogenetic movement which results in the formation of the AVE, have been proposed to move away from a region of high WNT activity [14]. Although β-catenin localization can be detected throughout VE, Wnt/β-catenin reporter strains generated to date have not exhibited activity at these early postimplantation stages making it difficult to determine the spatiotemporal dynamics of *in vivo *signaling within the VE.

**Figure 5:**
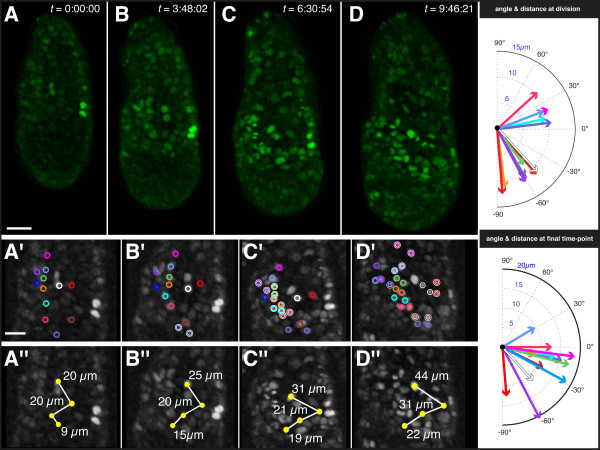
**Tracking H2B-GFP reporter expressing cells in the visceral endoderm of an E5.5 *TCF/Lef:H2B-GFP *embryo**. Rendered images of 3D time-lapse data of E5.5 *TCF/Lef:H2B-GFP *embryo acquired on a spinning disc confocal (A-D), with high magnification detail in greyscale shown beneath (A'-D' and A'' = D''). Duration of time-lapse experiment was 9 hours 46 minutes and 21 seconds (*t *= 9:46:21). Individual cells identified by H2B-GFP nuclear-labeling were color-coded (open circles) and tracked using the spots function in Imaris (Bitplane, Inc.). First panel (A, *t *= 0) depicts the initial state, with lower panels depicting high magnification views of tracked cells and reference cells. At *t *= 3:48:02, (B) the first tracked cell division occurs and pushes the bottom-most reference cell to the left. Cell divisions are highlighted with a white outline on the color-coded open circles. In panel C, (*t *= 6:30:54) nearest-neighbor relationships are preserved subsequent to the near-synchronous division of five cells, despite substantial growth of the embryo. In the final panel, (D, *t *= 9:46:21) the non-dividing reference cells (yellow closed circles on lower series of panels) have shifted in their relative position and the distance between them has increased; however, the daughter cells produced by previous cell divisions along the left have maintained their nearest-neighbor relationships. The constriction in the range of angle between the cells, as well as the distance between reference cells, suggests that circumferential (lateral) expansion of the embryo is greater than the proximal-distal (longitudinal) growth. Scale bars: 30 μm, upper panel; 20 μm high magnification images, depicted in lower panels.

In *TCF/Lef:H2B-GFP *embryos GFP reporter expression was detected specifically in the VE tissue layer around the time of DVE specification (E5.5), and throughout the period encompassing AVE migration (E5.75). VE-specific localization was confirmed by colocalization with the pan-VE marker HNF4α [15]. GFP reporter expressing cells were not detected in the epiblast and extraembryonic ectoderm at this stage (Figure 4A, 4A', 4B, 4B'). Initially, only a subset of VE cells expressed detectable and variable levels of GFP expression. After initiation of AVE migration, the number of GFP-positive cells increased, although still exhibiting varying levels of fluorescence intensity (Figure 4B1, 4B'1; Figure 4B5- yellow arrowhead marks migrating DVE/AVE cells). A small group of cells that were robustly GFP-positive were initially observed in a broad region around the embryonic-extraembryonic junction (Figure 4 and 5). In the period leading up to the emergence of the primitive streak, in the majority of embryos analyzed, robustly GFP-positive cells were observed to be predominantly localized to the posterior side of the embryo, likely corresponding to the region of the posterior visceral endoderm (PVE) (Figure 4A1, 4D1, D3; white arrowhead). The localization of this cohort of GFP-positive was designated as putative PVE, since it was contralateral to Cer1-positive cells, which are localized anteriorly (Figure 4C3; white arrowhead). Cer1 antibody specificity in the DVE/AVE was confirmed by colocalization with GFP in *Hex:GFP *transgenic embryos (Figure 4C, 4C'; yellow arrowhead). Taken together these observations are consistent with previous reports suggesting a repulsive or counteractive effect of high levels of Wnt signaling on cells of the AVE [14]. Further in depth analyses will be required to determine the precise localization of Wnt-responsive cells, combined with a quantitative analysis of levels of reporter activity within the VE, during this critical period of embryonic development.

### Tracking TCF/Lef:H2B-GFP reporter expressing cells in the visceral endoderm

One of the advantages of H2B fusions as live imaging reporters is that they facilitate the identification and tracking of single cells while at the same time permitting visualization of an entire population. Indeed, how a group or population of cells can move collectively and in doing so radically change the structure and function of a tissue, is a central question in developmental biology, and underscores many integral morphogenetic cell behaviors driving embryonic development. Since the *TCF/Lef:H2B-GFP *transgenic is the first Wnt reporter line to reveal the dynamics of Wnt signaling activity in cells of the visceral endoderm of E5.5 embryos, we focused on this stage to study the behavior of cells expressing the reporter. We assumed that GFP-positive cells were either actively signaling, or had recently been transducing a Wnt signal and remained GFP-positive due to perdurance of the GFP protein (Additional file 1).

3D time-lapse movies of embryos pre-streak (PS) stage embryos (Figure 5, Additional file 1) confirmed that the number of GFP-positive cells increased within the VE as development proceeded, and confirmed the heterogeneity of GFP reporter levels among GFP-positive cells, as had been observed in sequentially staged embryos (Figure 4). Tracking of reporter-expressing VE cells in E5.5 embryos for over 9 hours, a period of time during which the AVE would have migrated, revealed extensive cell division (over 50% of cells tracked divided), and the conservation of nearest-neighbor relationships between GFP-positive cells. No change was observed in the relative position GFP-positive cells that were tracked, suggesting that the *TCF/Lef:H2B-GFP *reporter might not be labeling cells of the DVE/AVE.

In the data depicted in Figure 5, a total of 22 GFP-positive VE cells were identified and tracked. Color-coded open circles identify individual cells in Figure 5, and color-coded closed spheres identify individual tracked cells in Additional file 2. About half the tracked population (12 cells) divided during the 9 hours of time-lapse (Figure 5A - color-coded open circles with white outline indentify cells having divided since previous time-point shown). In nearly all cases, nearest-neighbor relationships were preserved both in regard to individual cells, as well as their relative positions within the group.

We selected 4 cells that did not divide during the time-lapse as a reference set (closed yellow circles in Figure 5, yellow spheres in Additional file 2), and documented their relative distances during the period of the time-lapse (white lines connecting yellow circles in Figure 5A, red lines connecting yellow spheres in Additional file 2). Notably, the distance between these non-dividing reference cells doubled as their individual positions changed relative to each other. Despite this fact, their nearest-neighbor relationships remained predominantly unchanged, as did their position relative to their neighbors, suggesting that the topology of cells within the VE was constant.

We tracked dividing reporter-expressing VE cells. We plotted the orientation of the division planes, as well as the final position of daughter cells at the last time-point for which we generated image data (Figure 5). Our data suggest that any migration of reporter-expressing cells may result from oriented divisions, as well as a general increase in the size of the embryo, rather than by an active movement of cells. However, additional data and statistical analyses will be required to determine if cell divisions within this population exhibit a prevalent orientation, as our data might suggest.

As reporter-expressing VE cells proliferated, their progeny did not reorganize, but retained their relative positions. A minor reorganization or 'jostling' of cells could serve to alter cell geometry, but facilitate conservation of cell topology, within the VE epithelium and could be driven by differential regional proliferation within the VE. Our data support a model whereby active cell migration driving AVE formation [16], and cell proliferation may together result in a reorganization of cells within the VE epithelium. These morphogenetic cell rearrangements are also likely influenced by, and are expected to accommodate, the rapid growth of the embryo, notably the adjacent epiblast, at this stage. Further detailed analyses will be important in extending these observations and determining the respective roles of proliferation and cell signaling within the VE and its neighboring tissues, and importantly how these coordinated cell behaviors in association with cell signaling might direct the morphogenesis of the early mouse embryo.

### The TCF/Lef:H2B-GFP reporter marks the primitive streak and nascent mesoderm at gastrulation

Gastrulation is the event that results in the generation of the three primary germ layers (ectoderm, definitive endoderm and mesoderm) from the pluripotent epiblast, and the elaboration of the axes (anterior-posterior, dorsal-ventral and left-right). In the mouse, the onset of gastrulation is marked by the appearance of the primitive streak (the source of mesoderm and definitive endoderm) which represents a morphologically-distinct structure which breaks the bilateral symmetry of the epiblast, and in doing so, defines the posterior of the embryo at E6.5. The site of primitive streak formation has been proposed to be regulated by Nodal and Wnt3 signaling activities likely emanating from the overlying visceral endoderm [17-20]. This data combined with the expression pattern of *Wnt3 *suggest an essential role for Wnt signaling in primitive streak specification, and the maintenance of gastrulation. By E7.5 *Wnt3 *is downregulated while the related gene, *Wnt3*a, is activated. Embryos lacking *Wnt3a *exhibit a complete absence of paraxial mesoderm, the cell type emerging from the primitive streak starting at E7.5. These genetic data support an essential role for Wnt/ß-catenin signaling associated with the site of the primitive streak, in the initiation and progression of gastrulation in the mouse.

**Figure 6:**
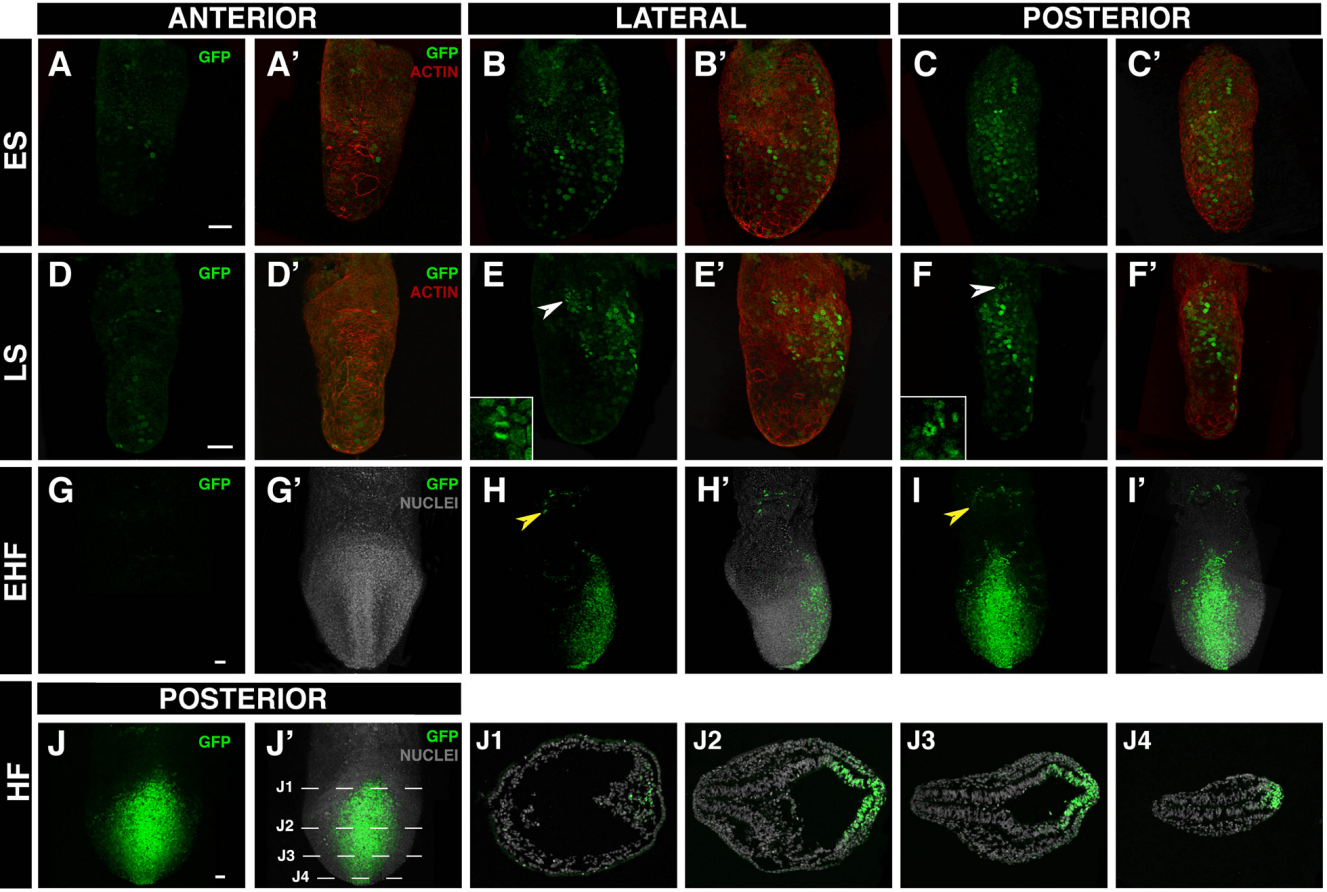
***TCF/Lef:H2B-GFP *reporter expression in gastrulating embryos**. Laser scanning confocal images of gastrulating embryos from early streak (~E6.5) to early heafold stages (~E7.75). Anterior (A, A'), lateral (B, B') and posterior views (C, C') of an early streak *TCF/Lef:H2B-GFP *embryo counterstained for actin. Anterior (D, D'), lateral (E, E') and posterior views (F, F') of a late streak embryo counterstained for actin. Anterior (G, G'), lateral (H, H') and posterior views (I, I') of an early headfold embryo counterstained for cell nuclei. Posterior view (J, J') of a headfold embryo and sections (J1-J4) through it along the indicated planes showing GFP expression in the streak and ingressing mesodermal cells. White arrowheads point to mitotic figures. Yellow arrowheads mark blood islands of the yolk sac. Scalebar: 50 μm. ES, early streak; EHF, early headfold; HF, headfold; LS, late streak.

To this end, *TCF/Lef:H2B-GFP *reporter expression reproduced *Wnt3 *early expression in the proximal epiblast before the onset of gastrulation (pre-streak (PS) stage, ~E6.0) and later on, in early streak (ES, ~E6.5) stage embryos, became restricted to the posterior side, where single cells within the epiblast became positive for the reporter and could be visualized as they underwent gastrulation traversing the primitive streak (Figure 6A-6C and data not shown). At this stage *TCF/Lef:H2B-GFP *reporter expression was restricted to cells of the primitive streak. By late streak stages (LS, E6.75), the level of streak-specific expression of the H2B-GFP reporter increased, and by the time the streak had fully elongated and reached the distal tip of the embryo, H2B-GFP fluorescent cells could be seen along the posterior body axis (Figure 6D-6F). Tissue sections of gastrula stage (E7.75) embryos, revealed the presence of individual H2B-GFP-positive cells within the epiblast in the vicinity of (and likely to be ingressing through) the primitive streak, as well as in the wings of mesoderm migrating away from it (Figure 6J).

By late bud/early headfold (~E7.5) stages, a second population of H2B-GFP-positive cells located in the proximal part of the conceptus within the extra-embryonic region emerged. These small patches of H2B-GFP-positive cells resembled the pools of primitive erythroid cells, or "blood islands", a site at which Wnt signaling has been proposed to play a role in specifying hematopoietic cell populations (Figure 6H, 6I; yellow arrowhead) [21-23].

### TCF/Lef:H2B-GFP reporter during midgestation

**Figure 7:**
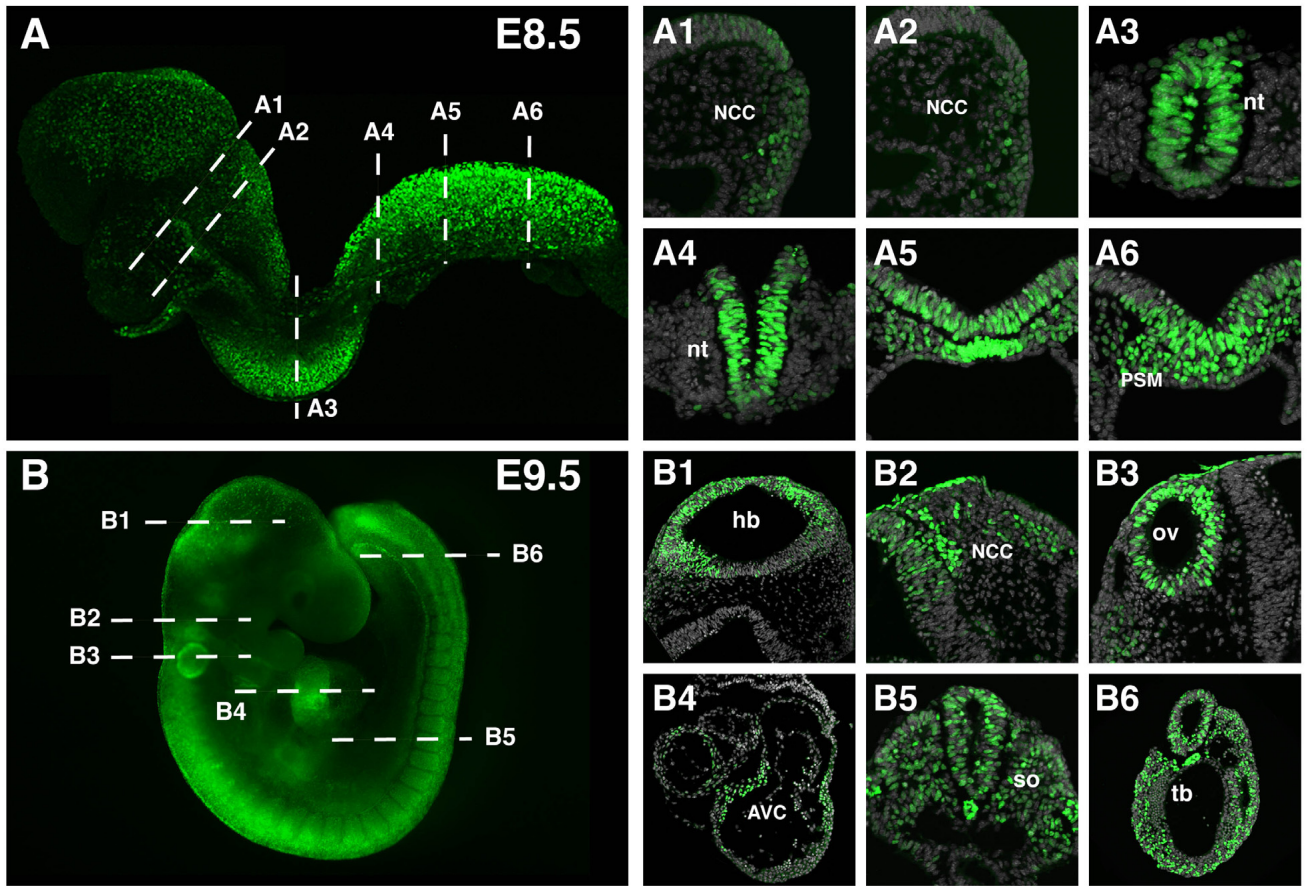
***TCF/Lef:H2B-GFP *reporter transgene expression in E8.5 and E9.5 embryos**. (A) Laser scanning confocal image of an 8-somite embryo and sections through it showing expression in the neural crest cells (A1, A2), the neural tube (A3, A4) and the presomitic and node region (A5, A6). (B) Widefield fluorescent image of a 23-somite stage embryo. Transverse sections reveal transgene expression in the brain (B1), the neural crest cells (B2), otic vesicle (B3), heart (B4), somites, neural tube (B5) and tail bud (B6). Aproximate planes of section are depicted by dashed lines. AVC, atrioventricular canal; hb, hindbrain; NCC, neural crest cells; nt, neural tube; ov, otic vesicle; PSM, presomitic mesoderm; so, somite; tb, tail bud.

By the time embryos had developed seven to nine somites (E8.5) robust levels of transgene expression were evident along the presomitic mesoderm and newly formed somites (Figure 7A, 7A5, 7A6). This strong posterior reporter expression might reflect the activity of *Wnt8a *and *Wnt3a *which are highly expressed at the streak at these stages, the last being required for mesoderm formation and somitogenesis [24-26]. By this stage, additional sites of the H2B-GFP reporter could be detected including the posterior neural plate, which displayed high levels of transgene expression. However, levels of reporter expression decreased in the anterior neuroepithelium (Figure 7A3, 7A4). In the head and branchial arch region, H2B-GFP expression was detected in cranial neural crest cells at the neural folds, as well as in cells migrating into the face and branchial arches (Figure 7A1, 7A2). Indeed, the generation of multipotent neural crest cells has been long associated with early induction through exposure to Wnt signaling [27].

### TCF/Lef:H2B-GFP reporter expression at midgestation to later fetal stages

**Figure 8:**
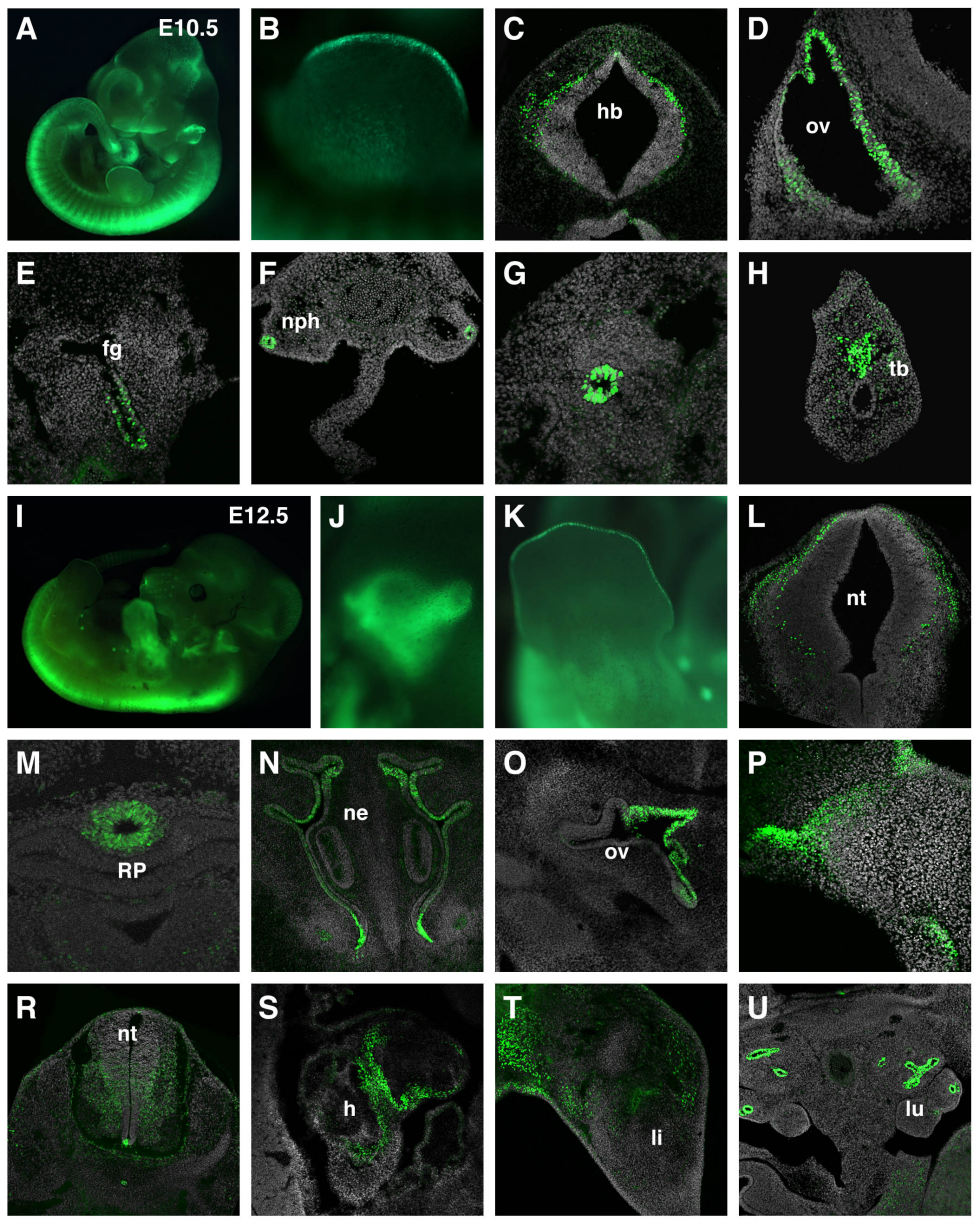
**Reporter transgene expression during midgestation**. (A) Widefield fluorescent image of an E10.5 embryo. (B) Restricted GFP expression in the AER. Bright expression is detected in individual cells in the hindbrain (C), the otic vesicle (D), the foregut endoderm (E), the lining of the mesonephric duct (F, G) and the tailbud. Widefield fluorescent image of an E12.5 embryo (I) and close-ups of the ear (J) and limb (K). Transverse sections at E12.5 reveal transgene expression in the hindbrain (L), the infundibulum (M), the olfactory epithelium (N), the otic vesicle (O), oral ectoderm (P), spinal cord (R), heart (S), limbs (T), lung epithelia (U). fg, foregut; h, heart; hb, hindbrain; li, limb; lu, lung; ne, nasal epithelium; nph, mesonephric duct; nt, neural tube; ov, otic vesicle; RP, Rathke's pouch; tb, tail bud.

At E9.5 transgene expression persisted in all previously described domains (Figure 7B1, 7B2, 7B5, 7B6). At this stage, expression in the somites reached more anterior levels and increased fluorescence could be seen all along the neural tube, also in the brain. New sites of expression were observed in the otocyst (Figure 7B3), and in restricted regions of the heart tube like the atrioventricular (AV) canal (Figure 7B4). At E10.5 *GFP *expression was maintained in the neural tube, somites, heart, nasal and pharyngeal region (Figure 8A, 8C). Expression in the otocyst was restricted to the dorsomedial epithelium, where Wnt signaling together with Shh have been shown to regulate dorsal-ventral axis specification (Figure 8D) [28]. By this stage *H2B-GFP *expression in the tail bud became restricted to a defined population (Figure 8H). In the limb bud, individual H2B-GFP-positive cells within the apical ectodermal ridge (AER) were first seen at this stage (Figure 8B). Expression of different Wnt ligands such as *Wnt3a *or *Wnt6 *in the AER has been previously reported, further confirming the specificity of the reporter as a Wnt signaling readout [29-31]. Positive cells were detected in the foregut endoderm, possibly corresponding to lung progenitors (Figure 8E) [32], but requiring further confirmation by marker analysis. In addition, the epithelial cells that form the lining of the mesonephric duct were also *H2B-GFP *positive as seen in transverse sections at E10.5 (Figure 8F, 8G). *Wnt9b *is expressed in the duct and is essential for early urogenital system organogenesis supporting a role for Wnt/ß-catenin signaling in this process [33].

Widefield fluorescence imaging at E12.5 revealed high levels of reporter expression within the spinal cord and limbs (Figure 8I). In the brain, expression of the transgene was localized to discrete regions such as the infundibulum, whereas it was broader in the spinal cord (Figure 8M, 8L, 8R). Close-up of the otic region showed increased levels of fluorescence in the developing semicircular canals (Figure 8J). In sections through the inner ear, transgene expression was detected in the dorsomedial otic epithelium that will give rise to the vestibular structures (Figure 8O). Previous studies have also described Wnt activity in the olfactory epithelium (Figure 8N) and oral epithelium (Figure 8P) [34]. High magnification wholemount views of the limb revealed continued expression of the transgene at the AER (Figure 8K), while sections showed regions positive for the transgene outside cartilage primordia (Figure 8T). Moreover, high levels of transgene were also detected in the lung epithelia (Figure 8U), where Wnt signaling is proposed to play a key role in branching morphogenesis [35].

### TCF/Lef:H2B-GFP reporter expression at postnatal stages

**Figure 9:**
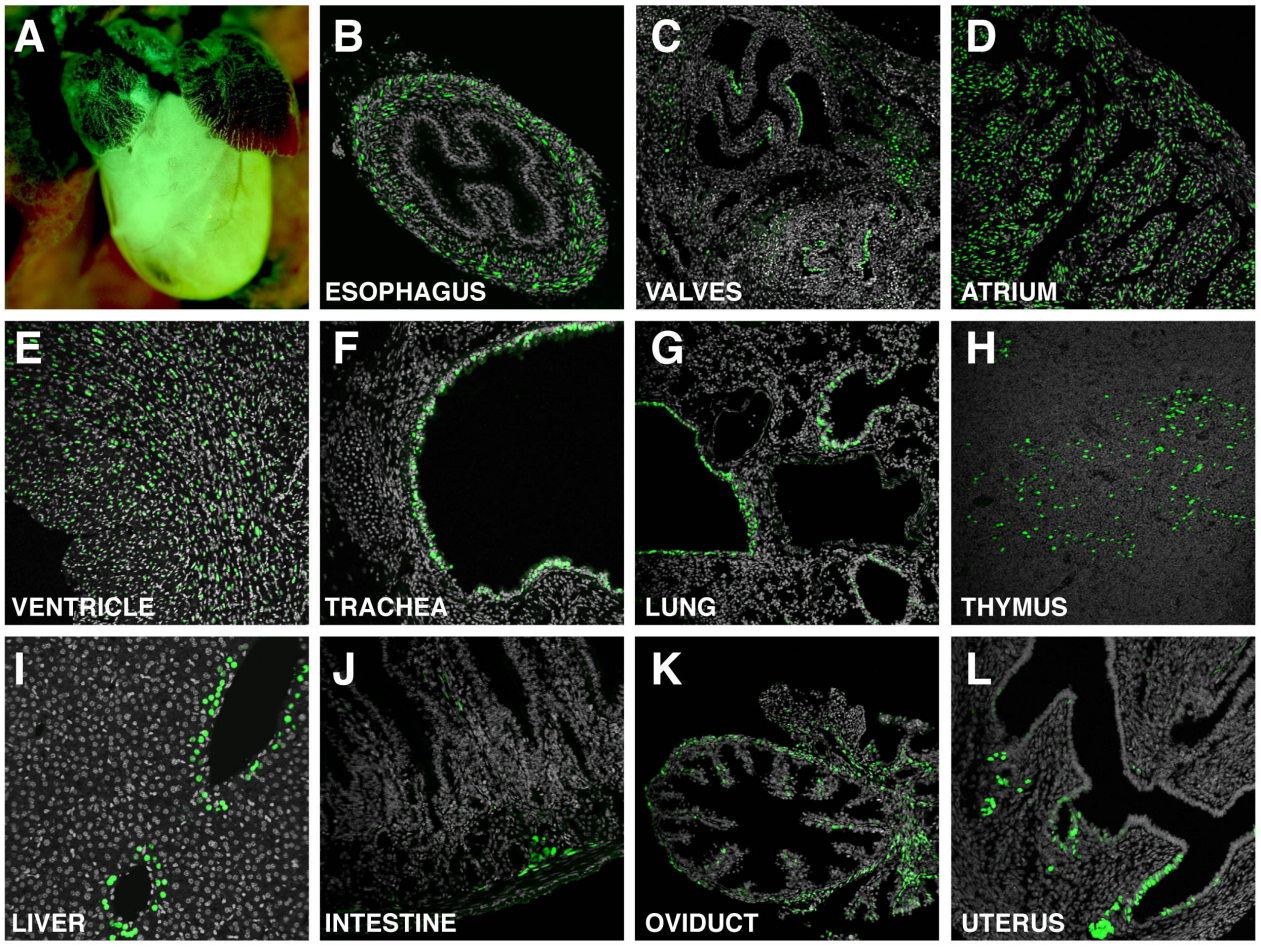
***TCF/Lef:H2B-GFP *expression in postnatal stages**. (A) Widefield fluorescent image of a P2 heart. Restricted expression of the transgene is detected in the esophagus (B) and heart valves (C) at P2. At P21, cell-type specific transgene expression is seen in the atrium (D) and ventricle (E) of the heart, tracheal epithelium (F), lung (G), thymus (H), liver (I), intestine (J), oviduct (K) and uterus (L).

To further validate the specificity and utility of the reporter, *TCF/Lef:H2B-GFP *transgene expression was also analyzed at postnatal stages. Robust but restricted reporter expression was observed in many tissues, with only a few documented in detail for this report. At postnatal day 2, H2B-GFP fluorescence was observed in the muscle layer or muscularis externa of the esophagus (Figure 9B). By three weeks of age, almost widespread expression of the transgene was detected in the atrial and ventricular myocardium of the heart (Figure 9A, 9D, 9E). However, restricted expression of the transgene was observed in the heart valves (Figure 9C). In the respiratory tract, reporter expression was restricted to epithelial cells in the lining of the trachea (Figure 9F), bronchi and bronchioles (Figure 9G) as previously described for a LacZ based Wnt/ß-catenin signaling reporter [36]. In the thymus, scattered positive cells were observed in the thymic medulla (Figure 9H), supporting a recent report demonstrating the role of Wnt signaling in epithelial microenvironment maintenance [37]. While in the liver, H2B-GFP fluorescent hepatocytes were only located in a single-cell layer surrounding the central veins (Figure 9I). This hepatocyte subpopulation was also identified using a different GFP-based Wnt/ß-catenin signaling reporter [7]. In this study these reporter-positive cells were shown to co-label with glutamine synthase, a marker for perivenous hepatocytes [7]. In the small intestine, *H2B-GFP *reporter expressing cells were primarily located in the crypts where they have been shown to represent the progenitor cell population of the intestine (Figure 9J) [38]. In the female reproductive tract, expression of the transgene was found in distinct layers of the oviduct including the muscularis and the lamina propia within the mucosa (Figure 9K). In the uterus, GFP reporter expression was observed in the luminal epithelium (Figure 9L). This observation is in agreement with reports identifying the Wnt/ß-catenin pathway as a major signaling pathway involved in embryo-uterus cross-talk during implantation [39,40].

### TCF/Lef:H2B-GFP reporter expression in the kidney

**Figure 10:**
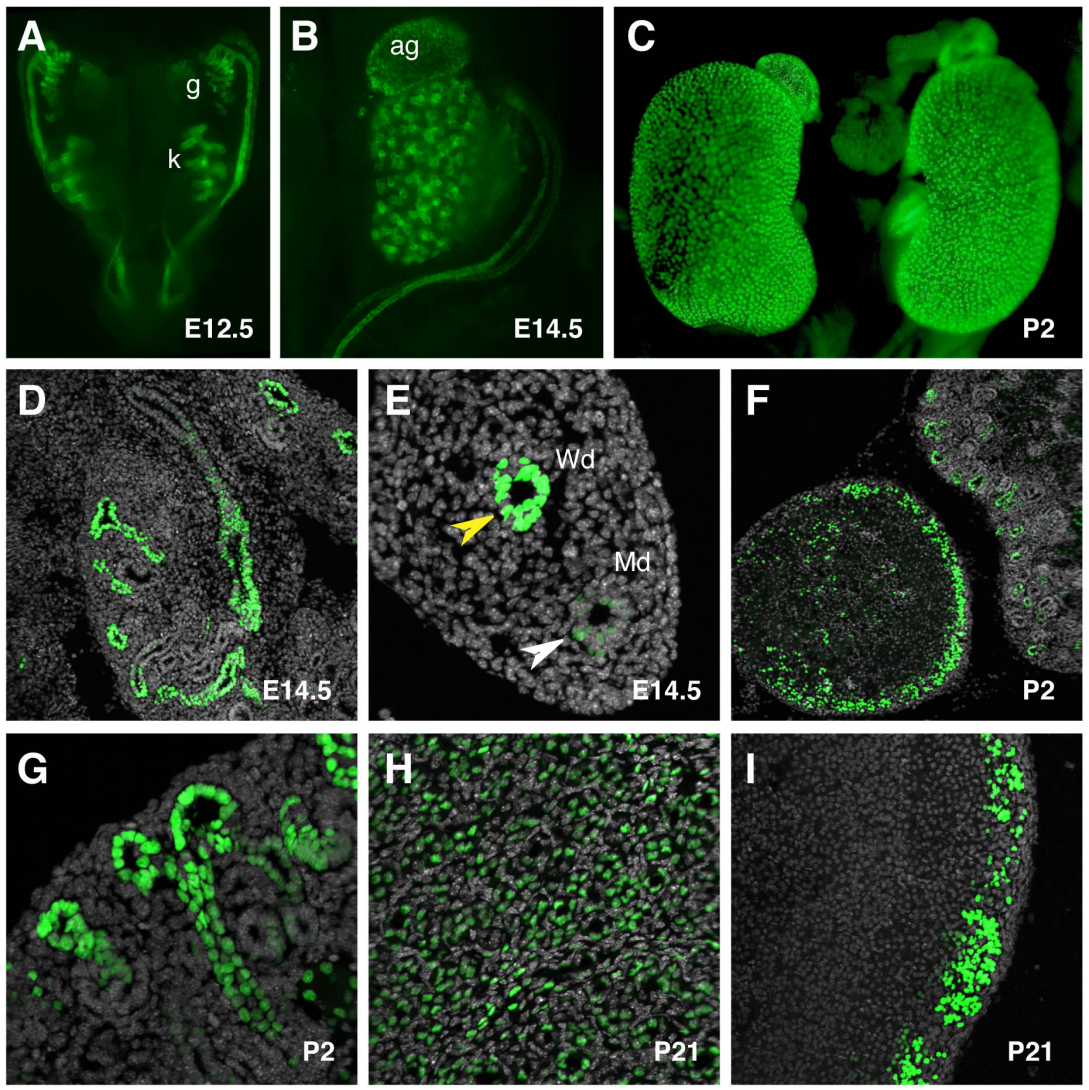
**TCF/Lef:H2B-GFP reporter gene expression during kidney development**. (A) Widefield fluorescent dorsal view of the urogenital tract at E12.5. Widefield fluorescent view of the kidney and suprarenal gland at E14.5 (B) and P2 (C). (D) Expression in the kidney is restricted to the collecting ducts. (E) Mesonephric and paramesonephric ducts positive for the transgene at E14.5. (F) Transverse section through the suprarenal gland showing high levels of the transgene in the cortical region at P2. (G) Detail of the renal cortex at P2 showing a branching event. High magnification images of the kidney (H) and adrenal cortex (I) at P21. Yellow arrowhead marks Wolffian duct, white arrowhead marks Müllerian duct. ag, adrenal gland; g, gonad; k, kidney; Md, Müllerian duct; Wd, Wolffian duct.

To validate the *TCF/Lef:H2B-GFP *reporter as an improved tool for visually dissecting morphogenetic processes driven by Wnt/ß-catenin pathway in any developmental event, we focused on the development of two major organ systems; the kidney and the brain. The mammalian kidney develops from a reciprocal signaling interaction between the epithelial ureteric bud and the adjacent metanephric mesenchyme [41]. By E12.5, distinct expression of the *H2B-GFP *reporter was observed in the urogenital anlage, where the developing kidneys as well as the gonads were positive for the transgene (Figure 10A). By contrast the mesonephros was negative for the reporter. In the kidney, Wnt reporter expression was restricted to the epithelial cells forming the collective ducts, as observed in both whole mount and transverse sections of an E14.5 kidney (Figure 10B, 10D). Differential expression of the *H2B-GFP *transgene was observed in the Wolffian and Müllerian ducts. In the mesonephric or Wolffian duct, reporter levels were higher than in the Müllerian duct (Figure 10E). At E14.5, the adrenal gland was also positive for the transgene (Figure 10B). At postnatal stages, expression of the reporter persisted in the adrenal gland, primarily in the zona glomerulosa of the adrenal cortex as evident in transverse sections (Figure 10C, 10F, 10I). Cortical expression of the transgene was in agreement with previous reports highlighting a requirement for Wnt signaling in the region for proper hormone production [42]. Also at postnatal stages, H2B-GFP fluorescence was detected in the collective ducts of the kidney (Figure 10G, 10H). The single-cell resolution of the reporter facilitated the detailed visualization of branches in the renal cortex (Figure 10G). This resolution of reporter if coupled with 3D time-lapse imaging of ureteric bud explants should facilitate the detailed analysis of cell behaviors driving branching morphogenesis [43].

### TCF/Lef:H2B-GFP reporter expression in the brain

**Figure 11:**
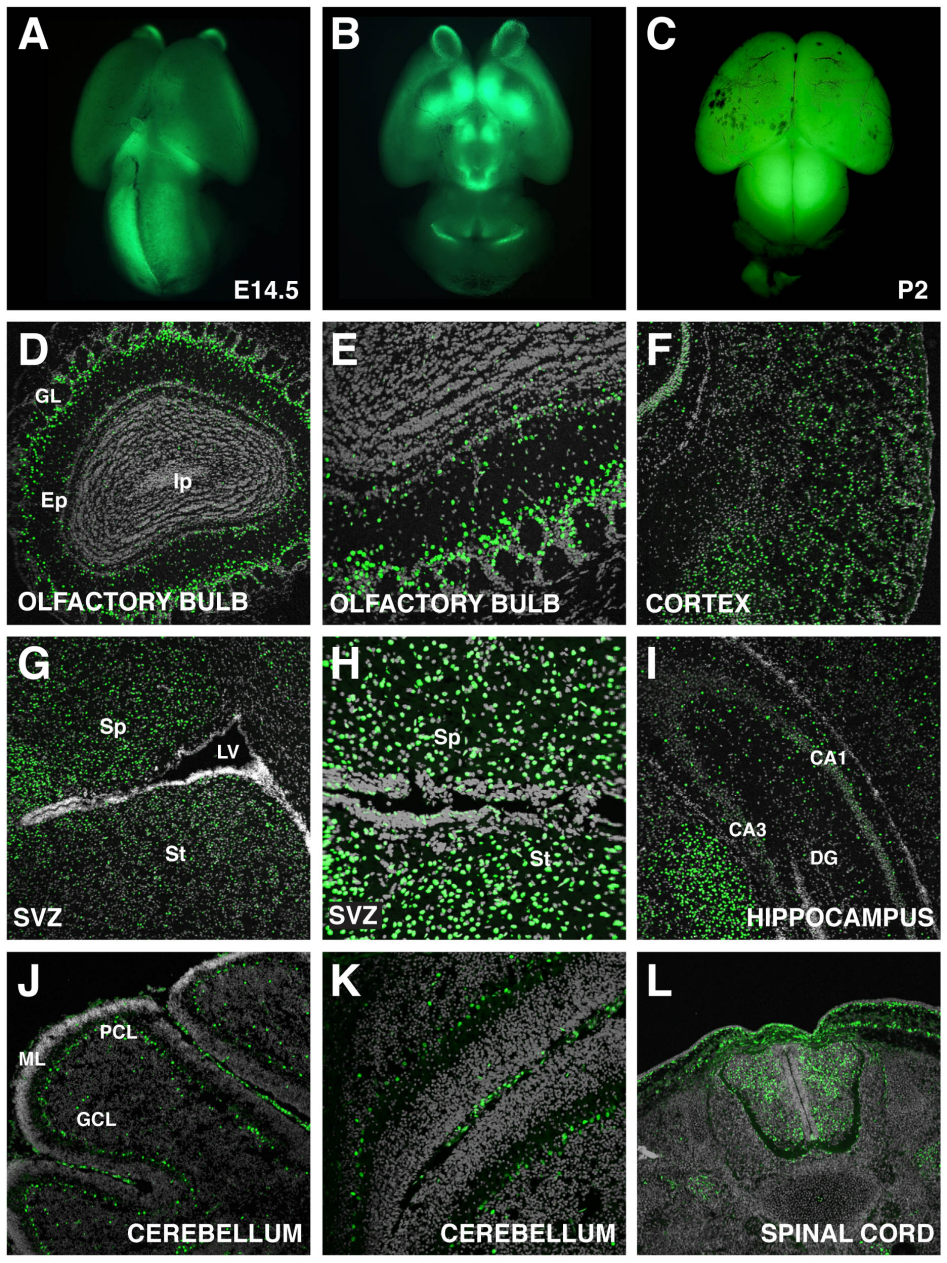
**Reporter transgene expression in the brain**. Widefield fluorescent dorsal (A) and ventral (B) views of an E14.5 mouse brain. (C) Dorsal view of a P2 mouse brain. Section through the whole olfactory bulb (D) and detail (E) showing GFP expression mostly in the periglomerular cells at P21. (F) Widespread transgene expression in the cortex at P9. Sections through a P21 brain showing GFP fluorescence in the septum, striatum (G), ventricle (H) and hippocampus (I). (J) Sagital section through a cerebellar lobe and detail of a fissure showing restricted expression at P9. (L) Transverse section through the spinal cord at E14.5. DG, dentate gyrus; Ep, external plexiform layer; GCL, granule cell layer; GL, glomerular layer; Ip internal plexiform layer; LV, lateral ventricle; ML, molecular layer; PCL, Purkinje cell layer; Sp, septum; St, striatum.

*TCF/Lef:H2B-GFP *transgene expression was also analyzed in the brain. During embryonic stages, reporter fluorescence was primarily localized to the olfactory bulbs, mesencephalon and part of the telencephalon (Figure 11A, 11B). At postnatal stages, widefield fluorescent views of the brain revealed increased levels of fluorescence (Figure 11C). However, when examined at high magnification, transgene expression was shown to be restricted to specific neuronal subtypes in different brain regions. In the olfactory bulb, tufted cells in the external plexiform layer and periglomerular cells were positive for the transgene (Figure 11D, 11E). These periglomerular cells might correspond to olfactory ensheathing cells, which have been previously reported as Wnt signaling responsive cells [44]. H2B-GFP fluorescence was widely observed in the cortex (Figure 11F). The transgene was also broadly expressed in the septum and striatum around the subventricular zone (SVZ) (Figure 11G). Section through the SVZ confirmed positive cells that colocalized with GFAP, a marker of SVZ niche astrocytes (Figure 11H and data not shown). Reporter fluorescence was also detected in the astrocytes of another neurogenic zone in the adult, the hippocampus (Figure 11I and data not shown), where a previous study demonstrated a role for Wnt signaling in the regulation of adult neurogenesis [45]. In the cerebellum, H2B-GFP expression was exclusively restricted to the Purkinje cell layer where *Wnt3 *is expressed in the adult brain (Figure 11J, 11K) [46]. Analysis of the peripheral nervous system was only performed at developmental stages where *TCF/Lef:H2B-GFP *expression was broadly detected in the spinal cord (Figure 11L). The sensitivity, resolution and localization of the *TCF/Lef:H2B-GFP *reporter if combined with time-lapse imaging of brain slice cultures should facilitate the detailed analysis of cell dynamics driving a wide variety of processes including neurogenesis.

## Conclusions

We have generated a single-cell resolution fluorescent transgenic Wnt reporter strain of mice and have performed an in depth characterization of its expression in embryos and several postnatal stages. The reporter design comprises multimerized TCF/Lef DNA binding sites driving expression of H2B-GFP, a subcellularly-localized fusion of a genetically-encoded fluorescent protein to human histone H2B, which by labeling active chromatin enables the visualization of single cells within a cohort. H2B fusion reporters not only facilitate the visualization and tracking of labeled cells over time, but also provide spatiotemporal information on cell proliferation and apoptosis dynamics. Moreover, to test canonical Wnt read-out efficiency of the *TCF/Lef:H2B-GFP *reporter line, we compared our line with the well characterized LacZ-based TCF/Lef-LacZ mouse strain, confirming specific GFP reporter expression in all expected sites of canonical Wnt signaling activity. Even thought Wnt reporter lines based on the multimerization of Lef/TCF binding sites which act as transcriptional reporters, might not be ideal readouts of active Wnt signaling, they are at present some of the best tools available to accurately identify the major sites of Wnt activity.

In conclusion, we demonstrate the specificity of the *TCF/Lef:H2B-GFP *transgenic line as a faithful readout of canonical Wnt signaling activity and its resolution at the single cell level. The increased brightness and sensitivity of the reporter has already revealed additional sites of reporter expression. While in readily detectable or robust sites of reporter expression reduced laser power is likely to be required for image acquisition, which is advantageous for live imaging applications. Since live imaging approaches critical for the study of cell dynamics, this reporter line represents a valuable tool to further our understanding of the events triggered by canonical Wnt signaling pathway activation during mouse development, tissue homeostasis or disease progression.

## Methods

### Generation of Reporter Constructs and Transgenic Animals

To generate the *pTCF/Lef:H2B-GFP *construct, six copies of the *TCF/Lef *response elements together with the *hsp68 *minimal promoter from the *TCF/Lef-LacZ *reporter construct [11] were inserted into the *Ase*I/*Nhe*1 sites of *pCMV::H2B-GFP*. Transgenic mice were generated by pronuclear injection following standard protocols. Animals were genotyped by PCR. Primers used for the PCR reaction were GFPGenotFW: ACAACAAGCGCTCGACCATCAC; GFPGenotRW: AGTCGATGCCCTTCAGCTCGAT. Two transgenic founder lines (TCF/Lef:H2B-GFP #16, TCF/Lef:H2B-GFP #61) were established both exhibiting similar patterns and levels of reporter expression. However, only one line (TCF/Lef:H2B-GFP #61) was further characterized in detail. Subsequent generations exhibited Mendelian transgene inheritance, stable transgene activity and comparable levels of reporter expression. Animals were maintained in accordance with National Institute of Health guidelines for the care and use of laboratory animals and under the approval of the Memorial Sloan-Kettering Cancer Center Institutional Animal Care and Use Committee.

### Embryo collection and strains used

Embryos were dissected in DMEM/F12 (1:1) containing 5% calf serum and fixed in 4% paraformaldehyde. Presomitic embryos were staged by morphological landmarks according to Downs and Davies [47]; somite stage embryos were staged according to their somite number. Additional mouse strains used were *TCF/Lef-LacZ *[11] and *TOPGAL *[6] reporters.

### Embryo culture

For *ex utero *culture embryos were dissected in DMEM/F12 (1:1) containing 5% calf serum and cultured in medium comprising 50% DMEM/F12 (1:1), 50% rat serum and 1% Penicillin/Streptomycin [48].

### Embryo sectioning

For vibratome sections, embryos were embedded 0.5% gelatin/15% BSA/20% sucrose (gelatin/albumin mix) and 4.5% glutaraldehyde in PBS and cut on a Leica vibrating microtome (VT1000) at 35-175 μm. For cryosections, samples were equilibrated in PBS/10% sucrose followed by PBS/30% sucrose overnight and snap-frozen in OCT (Tissue-Tek). Sections were cut in a Leica cryostat at 12 μm and counterstained with Hoechst for nuclei (5 μg/ml, Molecular Probes) and Phalloidin Alexa546 for F-actin (Invitrogen).

### ß-Galactosidase staining

Mouse embryos were dissected in PBS, fixed for 20 min in 0.35% glutaraldehyde in PBS and processed as whole mounts or embedded in the gelatin/albumin mix and sectioned. Samples were washed 3 times with X-Gal rinse (0.02% NP-40, 0.01% Sodium deoxycholate, 2 mM MgCl_2_, 100 mM sodium phosphate pH 7.3) for 20 min and incubated in 1 mg/ml X-gal, 5 mM K_3_Fe(CN)_6_, 5 mM K_4_Fe(Cn)_6_, 0.02% NP-40, 0.01% Sodium deoxycholate, 2 mM MgCl_2_, 100 mM sodium phosphate pH7.3 overnight. They were finally rinsed with PBS and fixed for 30 min in 4% paraformaldehyde.

### Immunostaining

Embryos were permeabilised in 0.55% Triton X-100 in PBS for 20 minutes and blocked in 10% fetal bovine serum in PBS for 1 hour. Primary antibodies used were: anti-Cer1 (R&D Systems), anti-Eomes (abcam), anti-GFP (Invitrogen), anti-HNF4a (Santa Cruz), and anti-Oct4 (Santa Cruz). Secondary Alexa Fluor (Invitrogen)-conjugated antibodies were used at a dilution of 1/500. DNA was visualized using Hoechst 33342 (5 μg/ml, Molecular Probes).

### Image acquisition

Widefield and epifluorescence images were acquired using a Zeiss Axiocam MRc camera coupled to a Leica M165FC dissecting scope or a Zeiss A1 Axioscope. Laser scanning confocal data were acquired using a Zeiss LSM510 META using a PlanApo 20×/NA0.75 objective. Fluorophores were excited using a 405 nm diode laser (Hoechst), 488 nm Argon laser (GFP, GFP) or 543 nm HeNe laser (Alexa 546). Embryos were imaged whole mount in MatTek dishes (Ashland). Spinning disc confocal 3D timelapse data was acquired using Volocity acquisition software http://www.improvision.com/ and a Perkin-Elmer RS3 Nipkow-type scan head mounted on a Zeiss Axiovert 200 M with Hamamatsu C4742-80-12AG camera. GFP was excited using a 488 nm Argon laser. Images were acquired using a Zeiss plan-Neofluar 25×/0.8 DIC korr objective. 10-20 *xy *planes were acquired, separated by 3-4 μm. Time intervals between *z*-stacks were 15 minutes. For live imaging experiments, embryos were maintained in a humidified, temperature-controlled chamber with 5% CO_2 _atmosphere. Sections were mounted in Fluoromount-G (Southern Biotech) and imaged through glass coverslips. Confocal images were acquired as *z *stacks of *xy *images taken at 1 μm z -intervals.

## Footnote

The Jackson Laboratory Induced Mutant Resource http://www.jax.org/imr/index.html will be distributing the *TCF/Lef:H2B-GFP *mouse line. The provisional reference name for the strain is: STOCK Tg(TCF/Lef1-HIST1H2BB/EGFP)61Hadj/J.

## Authors' contributions

AFV - carried out experiments to characterize the mouse *TCF/Lef:H2B-GFP *reporter strain, and wrote the manuscript. AP - carried out experiments to characterize the mouse *TCF/Lef:H2B-GFP *reporter strain. GT - carried out experiments to generate *TCF/Lef:H2B-GFP *constructs. RJA - performed image data analysis. DD - provided reagents making this study possible. AKH - conceived, designed, funded and supervised the project and wrote the manuscript. All authors critically read and revised the manuscript, and approved the final version.

## Supplementary Material

Additional file 1 **3D time-lapse movie of E5.5 *****TCF/Lef:H2B-GFP embryos***. Rendered images of 3D time-lapse data acquired on a spinning disc confocal (A-D) of a litter of embryos obtained from *TCF/Lef:H2B-GFP^Tg/+ ^*x ICR (wild type) cross. Half the embryos were hemizygous for the transgene. Embryos were dissected at E5.5 and exhibited variability in stage, which resulted in variability of the level of the fluorescent reporter and its localization. The time-lapse experiment was carried out for over 9 hours. Click here for file

Additional file 2 **Tracking H2B-GFP reporter expressing cells in the visceral endoderm of *TCF/Lef:H2B-GFP *embryos**. Registered rendered images focusing on a *TCF/Lef:H2B-GFP ^Tg/+ ^*embryo which remained in the field of view for the duration of the 3D time-lapse experiment depicted in the previous movie (A-D). Duration of time-lapse experiment was 9 hours 46 minutes and 21 seconds (*t *= 9:46:21). Individual cells identified by H2B-GFP nuclear-labeling were color-coded (colored spheres depict individual cells) and tracked using the spots function in Imaris (Bitplane, Inc.). The movie consists of 37 frames each depicting one time-point. The first frame (*t *= 0) depicts the initial state, with 4 non-dividing cells used as a reference for both position and distance. At frame 15 (*t *= 3:48:02), the first tracked cell division occurs below the bottom reference cell. Thereafter, tracked cell divisions occur in frames 21, 23, 25, 27, 28, 31, 33 and 37. Throughout the observation period, nearest-neighbor relationships are preserved, despite the substantial growth of the embryo. The change in the angle between the reference cells, as well as the distance between them, suggests that circumferential (lateral) expansion of the embryo is greater than the proximal-distal (longitudinal) growth. Scale bar: 30 μm depicted in lower left. Click here for file
